# When the Future is Not Bright: Social and Political Stakes in Discussing Childhood Cancer in Romanian Media

**DOI:** 10.3390/children6110126

**Published:** 2019-11-15

**Authors:** Adriana Teodorescu, Dan Chiribucă

**Affiliations:** Sociology Department, Babeș-Bolyai University, 400604 Cluj-Napoca, Romania

**Keywords:** death, children, childhood cancer, media, immanent transcendence, social transcendence, social illness, national illness, death acceptance

## Abstract

In contemporary societies’ perception of children, death plays an incredibly insignificant role. This role goes from being ornamental, a weak reminder that our civilisation has overcome the times of children’s high mortality rates, to being some other society’s concern. Despite both medical improvements and cultural constructions of the child as an immanent and social transcendence, children can and do die. Although an increasing number of recent studies disclose and legitimise children’s preoccupation with death and dying in the context of a popular culture fascinated with death, studies interested in the representations of death and dying in children are rather scant. In this article, we investigate the social and political stakes in discussing children’s cancer in today’s Romanian media, aiming to make visible how the illustrations of the connections between children, death and illness are never ethically neutral. We begin with the observation that, during recent years, there has been a growing media focus on childhood cancer in Romania. Adopting a qualitative approach and resorting to comparative analysis, we analyse what lies beneath the intentions of criticising troublesome socio-political or medical realities of childhood cancer, revealing the mechanisms through which childhood cancer is transformed into a social illness and the cultural implications for the acceptance of death as an inherent part of life both for children and the population as a whole.

## 1. Introduction

It might be more difficult now than ever before to discuss children and death. Advances in medicine, especially in the public health domain [1], which have taken place in the recent decades, including those that have eradicated or brought under control many infectious diseases through global vaccination, have substantially contributed to the decline of child mortality across the world [2,3,4]. Moreover, the average life expectancy has tremendously expanded since 1900, increasing by 5.5 years between 2000 and 2016 [5,6,7], with Romania being in line with this tendency [8,9,10]. In this context, all over the world, experts and scholars are discussing the phenomenon of the ageing population [11,12,13,14,15]. This demographic trend has contributed to making children appear to be social assets [3], by overlapping the idea of having and giving birth to children with the notion of a national continuity deemed to be increasingly ‘endangered’, while, on the other hand, senior citizens are beginning to be regarded as social burdens [16,17]. This socio-demographical rapport between a category that is excessive and one that is economically desirable but insufficient translates into personal codifications of cultural values. Thus, children are unprecedently viewed as personal forms of transcendence [18] (religious discourses often insist on this aspect as well [19]), pleasant means of achieving individual happiness [20] and important triggers of a sense of symbolic immortality—the sense of continuing beyond a personal life as a response to the fear of totally disappearing, unrelated to a specific religion [21,22,23,24]. From a socio-cultural perspective, children are becoming the great (and more and more improbable) solution for an ageing society, thus functioning as a form of social transcendence. Symbolic immortality through children, either on a personal or on a social level, is cultivated by a postmodern world anguished by and obsessed with the future [25]. In other words, at a deeper, metaphorical level, children are not only exempt from death but are potential carriers of its transgression. Childhood becomes the mirific, idealised realm [26], where stories of hope, growth and fantasy are told without the shadow of mortality [27]. At the same time, ageism is still, if not prevailing, undoubtedly prominent among the attitudes towards the elderly [16,28,29,30,31]. This hidden narrative menaces the discourse and human interactions, threatening social exclusion, which ultimately may lead to social death [32,33].

Slowly but steadily, all these elements have rendered less socially visible the manifold connections between children and death. The high mortality rate in children until the 20th century [2] and the perils to which they were exposed during their lives, including hunger, health problems, work and poor education as dominant dimensions for most [3,34], have started to fade away in the collective imagination. The death of children tends to be perceived more and more as an unnatural and exceptional phenomenon [35,36]. People usually feel uneasy mentioning death and children in the same sentence [36], and most of the social discourse considers any connection between the two inappropriate. This cultural reluctance also reverberates within the social sciences. Recently, what was called ‘the new sociology of childhood’ succeeded in giving academic legitimacy to childhood as a research theme that, unlike in previous studies, should be approached by taking into account the fact that children are endowed with agency, being social actors with their own value and specificity, unsubordinated to those of adulthood [37,38]. However, if death has found its way out from taboos and stigmata and given rise to the academic field of death studies [39], and if children are given much more attention as full subjects than a few decades ago [40], the same cannot be said about the two taken together as a research topic. Sarah Coombs [36] observes in her recent pioneering book combining death studies with childhood and youth studies, that within Western culture throughout history, death and children were in a very similar way both marginalised, feared and sometimes romanticised, but they were also secluded from one another. Coombs brilliantly explains that although today’s children are much less exposed to the possibility of dying and to witnessing non-mediated forms of death, they are artificially separated from it, in a utopic attempt of society to protect them from death as such. One of the main conclusions of Coombs’ book is that the dominant adult assumption that children do not, cannot and should not think about death is utterly absurd, because children do, with naturalness and creativity, and this is perfectly fine. 

Following Coombs’ suggestions, childhood and death could cohabitate as social realities and research topics, if children are allowed to express their own opinions and perspectives about death [36,40]. It is an excellent solution for bridging the social disconnection between death and childhood and for repairing the scholarly attitudes that have silenced or neglected the voices of children regarding death. However, what happens when it is not so much about how children understand death but more about how contemporary society understands the death of children when it is forced to confront the sad reality that, despite medical progress and family care, children still can and do die? Will society be able to offer coherent and culturally mature discourses, which would display a profound understanding of death as an inexorable human reality, as, using a Heideggerian terminology [41], a human being’s most ‘personal’ possibility? Would society be capable of accepting death without losing the positive values attached to the latest cultural constructions of childhood? Will it manage to maintain the balance between acknowledging the limits of any endeavour to fight death and the need to persist? It should be the role of social sciences to elucidate all these questions; however, the abovementioned poor intersection between research on death and research on children is a hindrance. Studies interested in the representations of death and dying in children are rather scant or lacking focus on social and political aspects [36]. When illness comes into play as the element responsible for the actualisation of the unfortunate possibility that ties together children and death, the analysis of social aspects is extremely important. In a world where, due to the remarkable growth in life expectancy [5,7], death is now associated with ageing [1,35], inducing the idea that the ‘normal’ cause for death is old age, there is hardly any possibly fatal illness that could escape the process of assigning social meanings. If death in old age is nature’s business, death at other ages is a matter of society’s responsibility. It is true that there are illnesses more ‘social’ than others—and this degree of ‘social’ is highly related to how socially visible an illness is—but it is certain that the social dimension of cancer is well known, already part of popular culture and common knowledge. Breast cancer, cervical cancer and lung cancer—they are all part of a greater narrative on cancer, society and death [42]. Childhood cancer constitutes, however, a bottleneck in this narrative, because it cannot be tamed by awareness and prevention campaigns and the fear of death lurks substantially more than in the other cases.

In the present article, we aim to contribute to research on death and children with a focus on the contemporary representations of childhood cancer by assuming a sociological point of view. More specifically, we explore the social, cultural and political stakes of discussing children’s cancer in contemporary Romanian media, aiming to reveal how illustrations of the relationship between children and death are never ethically neutral, especially when illness mediates this connection and even more so when the illness is cancer, doing much more than simply unravelling the contemporary and historical idealised images of a deathless, sacred childhood and a transcendent child. Although this study is focused primarily on Romanian representations, the analysis is set in a broader, international context.

## 2. Materials and Methods

This study is built on the constructivist premise that no reality can be taken for granted [43]. It also relies on the deconstructivist concept, according to which any social representation contains more layers of slippery meanings than the surface allows us to see [44]. Thus, even if social narratives revolving around childhood cancer are, in general, all over the world, articulated by the idea that children are significant sources of life meaning [45] and precious gifts for families and humanity [22,46,47,48] and these narratives are apparently committed to displaying and discussing dreadful socio-medical situations where children are exposed to death, they should be investigated as social constructions and, implicitly, as potential carriers of stereotypes, desirability biases, false information, manipulation and ideologies. We do not suggest that there is always or aprioristically a negative agenda behind these representations or discourses. More frequent are inaccurate or deformed visions of reality—the over-use of emotional scripts to the detriment of rational arguments. However, we do want to underline that, because these representations deal with such sensitive topics (cancer, children and death), they can furnish useful material regarding what our society values and apprehends.

The idea for this article arose when we became aware that, during recent years, there has been a growing media focus on childhood cancer in Romania, which we realised might have been (at least) partially engendered by a Romanian non-governmental organization (NGO)’s (The Give Life Association [49]) project to construct an oncological hospital dedicated exclusively to children. It was noticed that the topic became quite fashionable in the media, with an abundance of articles in newspapers and magazines and on the most well-known Romanian lay medical platforms, describing the main characteristics, symptoms, causes, prognosis and treatments of childhood cancer. The topic was also present in social media, with fundraising campaigns and shared photos of children with cancer. Stories related to childhood cancer were also very frequent in television news.

We decided to explore what may lie beyond the manifest layers of texts denouncing the social or medical realities of childhood cancer and to investigate the social, cultural and political aspects connected to how Romanian and Western societies perceive childhood, illness and death. Adopting a qualitative approach, we searched for materials and texts that were thematically relevant (i.e., having childhood cancer as the main subject and targeting a general audience) published online and on Facebook social networks, including news, lay medical articles, general articles, TV shows, and forums. 

We also carried out an in-depth analysis of the websites of the abovementioned social project, as well as those of three others that significantly joined the same cause or a similar one [50,51,52]. Google was used to conduct the searches, and a time filter was applied to identify results from 2000 to the present. There was a particular interest in materials dated after 2010–2011, as it was noticed that, before these years, the materials were less plentiful. Moreover, an increase in related media was observed in the last five years (being most pronounced in the last two years), along with a phenomenon that could be called ‘horizontal contamination’—the same content about childhood cancer was reproduced by numerous websites a short time after being originally published. The most relevant themes and topics identified in the analysed texts were compared with similar occurrences from other countries. Using this comparative framework, it was easier to refine what is distinctive in Romanian representations and to identify what could have cross-cultural relevance. It is also important to note that theories and concepts related to the death studies field were employed. 

## 3. Results and Discussions

One of the most pervasive and striking aspects that we noticed is that, although death is situated at the core of all of the texts dealing with childhood cancer, being a constant threat that articulates the discourse and shapes its content at the intersection between breaking news and tabloid titles (e.g., “More and More Children Are Suffering from Cancer”; “Find What Causes Our Children to Get Cancer” or “Our Children Are Dropping like Flies from Cancer”), it is referred to in surprisingly few verbal expressions. It is almost as if death is similar to an engine that puts a device into motion without being seen—something obvious yet impalpable. Taking this image further, it appears that death is unwittingly instrumentalised to function incompletely, not for itself, but prominently in life’s favour. 

This raised our interest in discussing at least the following topics: (1) the acceptance of death as a possibility of human being, in this case, of children, as the defining sign of finitude, of mortality understood as a normal life process, which philosophers distinguish from the idea of a timely existence [41,53] and that sociologists do not reduce to ageing [35,54]—rather, as can be conceived as an ontological datum; and (2) the scope of death denial [55,56] in representations that refer specifically to children and the social significances of both acceptance and denial [57] in a contemporary culture more obsessed with health than ever before [58]—a culture that valorises children and youth [59] and puts great emphasis on the personal and social benefits of parenthood [20,46,60,61,62]. It rapidly became clear to us that, if we succeeded in disentangling the intricate connections between children, death and illness, taking into account both the general Western traits and Romanian particularities, we might find some substantial answers to all these issues. 

### 3.1. Making Cancer a Social Illness

Known since antiquity, cancer—an abnormal rapid proliferation of cells that can develop in any organ and invade any part of the body—has been, since the nineteenth century, the name for more than 100 diseases that, besides sharing these characteristics, have their own traits in terms of causes, symptoms, treatment and prognosis. The second leading cause of death worldwide [63], inspiring negative reactions and sentiments—stigma directed to those suffering from the disease, fear, disgust, etc. [64]—cancer has achieved a social dimension during recent decades [42,65] in the sense that societies realised the need to actively fight against this threat, amongst others, by investing in medical and scientific research, by raising awareness and implementing politics of prevention and also by dismantling the stereotypes that have surrounded it. In sharp contrast with the infectious diseases that are now in stark decline due to antibiotic therapies [66], cancer is seen as a disease continuing to expand and to preserve its image as an incurable illness. Nevertheless, increased diagnoses because of more screening does not necessarily mean an increase in cancer mortality—quite the contrary. Recently, many forms of cancer have seen an improvement in terms of life expectancy prognosis [63]. 

#### 3.1.1. A Cultural Frame of Cancer’s Social Visibility

Apart from various medical interpretations, there is also a cultural explanation for the social perception of cancer as highly deadly and continuously increasing in number of victims. In Western societies, the cultural history of cancer went from social invisibility to social visibility, which should be understood in the sense of the cultural logics of visibility [67,68] and not as a sum of images of reality as such. Now, we have entered a different, new stage—that of ‘over-visibility’. The first phase lasted until the social dimension became palpable. Social invisibility refers, not only to the times when there were fewer discussions about and preoccupation with cancer, but also to the fact that it was considered a personal issue—the sole responsibility for being ill falling on the individual. Drawing ideas from her own experience as a cancer patient, Susan Sontag [65] was among the first social scientists who criticised two important processes. First was the psychologization of cancer that exempted society from taking any responsibility, blaming the individual for not being able to express their feelings and for lacking qualitative personal relationships and other similar elements that were deemed as good predictors of getting cancer. Secondly, Sontag rejected any form of metaphorization, of abusively enveloping cancer with layers of social, cultural and psychological meanings. Cancer is not the disease of the ‘other’; it is not a medical translation of capitalism. It is just a disease, with causes related to body and biology, and no one should feel ashamed of suffering from cancer. The second stage, that of social visibility, is the one that can be traced back especially to the beginning of breast cancer awareness campaigns, which have intensified and grown in number since 1990 [69], and to the recognition of the causal link between lung cancer and smoking cigarettes [70]. Within the social visibility stage, society was very often called into question regarding its capacity to guarantee that it did not spare efforts in order to fight the environmental and nutrition-related causes of cancer. The psychologization and metaphorization of cancer declined substantially during this phase, although they never disappeared, in particular, in popular culture, and in some circumstances, they proved to be quite vivid, such as when moderate psychosomatic approaches are meant to counteract the over-medicalisation of cancer [71].

The third cultural stage, social over-visibility, which should not be confused with the reality of cancer as being over visible, is shaped by the tendency to understand cancer as something that needs to be openly and intensely discussed. Cancer social visibility is more than recognising its social aspects; it is over-exposing them as a means to collectively fight against it—an attempt to exorcise it. Moreover, the third stage recovers parts of the psychologization typical of the first stage; cancer can be triggered by having a vulnerable personality or being exploited by society (e.g., too much work, stress) and the impulse for metaphorization (the military and martyric metaphors abound), which becomes even more obvious when comparing cancer to cardiovascular diseases. The social dimension of cancer, in this stage, is the one to be held most accountable in a sort of dichotomic relationship with the cancer patient. When it comes to childhood cancer, this is more applicable than in any other case, because the cultural imagery of a socially ‘wrong’ cancer meets the cultural imagery of innocence and because, as observed above, the association between old age and death leads to asking society to take responsibility for other forms of (possible) death in other age categories. Thus, a significant part of cancer’s over-visibility is marked by rage directed both against cancer as such (see, for example, the “Stupid Cancer” group on Facebook and the tones of intensively shared memes that begin with this formula) and against a society incapable of offering protection against it. According to one of the analysed articles, “cancer is a disease of whose onset makes us rebel against it. And this rebellion is infinitely greater when this merciless disease strikes a kid” [72]. 

#### 3.1.2. A Social Self-Inflicted Wound

This over-visibility of childhood cancer seems to characterise also the Romanian representations, especially the ones from recent years. The first indication of this over-visibility is contained in the many titles of articles from the media or from lay medical platforms that insist on the alarmingly increasing number of children that are suffering from cancer and on the onset of cancer at younger and younger ages, e.g., ”More and More Children Are Afflicted by Cancer” [73]; “400 Children Die of Cancer in Romania” [74]; “Worrisome Statistics Come from Constanța (*one of the largest Romanian towns*). The Number of Children Diagnosed with Various Forms of Cancer Almost Tripled in Only One Year” [75]; “Why Our Children Are Dropping Like Flies. The Survival Rate in Romania Is 50%, Much above the European One” [76]. Similar observations regarding a number, which is seen as continuously expanding and encompassing more childhood ages and is being characterised by a growing aggressivity, can be seen in the articles’ content as well, e.g., ”this disease is more and more aggressive and it strikes at younger and younger ages”; “the oncologists discuss about more and more cancers” [73]; “the statistics are alarming” [75]; “Pediatric cancer—besides an increasing number of diagnostics—is also on the rise in what concerns the aggressivity of its manifestations: multiple simultaneous sources of disease, multiples organs affected at the same time and a period of spreading much reduced compared to what was described until now in medicine books” [77]. Other indicators of such over-visibility are the celebrities and other famous Romanian people who promote, inclusively through short videos, the Give Life Association’s project. This situation is depicted very positively by articles, TV news and especially by the NGO’s webpage: “Prestigious personalities from Romanian public space drawing attention to a terrifying reality: the lives of Romanian children with cancer are hanging on a thread” [78].

It is not our goal here to factcheck the medical validity of all these claims; it would be difficult, as Romania possesses no exact data regarding cancer [79] and still insufficient data concerning childhood cancer [80]. What is certain is that these discourses play with numbers to obtain an emotional response from audiences more than to convey a clear message. For example, saying that the cases *tripled* (see the quote above) elicits more anxiety than simply saying what the article discloses in the end: “from 11 cases in 2015 it went to 30 in 2016”. This is quite interesting if we think that numbers belong rather to the realms of facts and data rather than to the sphere of emotions and sentiments. Actually, beyond choosing to represent the reality of childhood cancer through one number or another, the analysed texts tend to depict, through a process of over-generalisation [81], childhood cancer as an impending reality—as a calamity bound to happen to many children. They do this by appealing to ad verecundiam (the argument from authority) [81], referring multiple times in the same article/news/story to experts and specialists to bolster their claims. There is hardly a single (news) article in which experts and specialists are missing and, interestingly, very few in which the words “doctors” or “physicians” are mentioned. This appeal to experts can also be encountered in the media from other Western countries, but studies and specific names of specialists are indicated [82]. 

Being interested in putting these findings in a broader framework, we searched on the internet for recent articles related to childhood cancer published in the United Kingdom (UK), United States of America (USA) and France. The first results returned were not magazine or news articles but links to the websites of different cancer associations (Cancer Research UK for the UK; American Cancer Society for the USA; Enfants sans Cancer for France), where there is much more structured information and the language relies less on the emphasis on children as being in danger of getting cancer just by being children and also less on the alarmistic rhetoric present in the Romanian discourses. This also applies to the articles that we found in these countries: “Survival rates improve across the globe (…) Childhood cancers have risen across the globe by 13% over 20 years, according to data from the World Health Organization’s cancer section. (…) Part of the reason for the rise is thought likely to be better detection” [83]. However, we did a wide search, and there may be cases in which the discourse could be similar to that in articles from Romania (in rather tabloid-oriented media). However, the existence of a more objective prominent discourse says a lot about the differences between societies in approaching childhood cancer. 

With few exceptions (e.g., the articles from the MedLife medical platform, which adopts a more neutral, objective tone [84] or Doctorul Zilei/The Doctor of the Day [85]), these articles, be they from newspapers, magazines, internet websites or broadcasted on news stations, contain two ways of understanding visibility and are reminiscent of the first and second stages of cancer’s cultural framework. First, they suggest that there is a poorly understood visibility of cancer. That is, cancer manifests within the world of children without being properly understood, with the consequence, as they claim, that many parents and doctors do not recognise the signs of cancer. This insufficient visibility is the visibility of signs, of hidden messages of cancer, and it needs to be changed into the good, action-oriented visibility. Society’s part is more important here, in this second visibility, but also in the connection between the two, because it needs to find tools to interpret the signs, helping parents to read them and, more importantly, to cure them. As there are not too many forms of medical prevention in the sense that, unlike adult cancer, there is nothing a child can do to avoid getting sick (a fact stressed by almost all discourses), society becomes a sort of scapegoat. It is to be noted that there are some representations in which parents, especially mothers, are indirectly held accountable for children’s cancers, mostly regarding genetic causes (e.g., children with mothers who have breast cancer at a young age could be at risk for sarcoma), but they are rather rare [86,87], while in the vast majority of our materials, the blame is on society as a whole and not on mothers, who are rather incorporated into children’s suffering. 

Society, in general, is considered responsible for childhood cancer in Romanian narratives when those narratives seem to convey an impossibility, inability or unconcern to designate more specific units or social levels, such as the political system or the medical system, or to gravitate around a Romanian national specific. Although these units or levels are somehow implied, the focus remains on society as such. The articles outline several culprits that are understood to be expressions of a society incapable of taking care of its inhabitants, such as the environment with its pollution, the growth hormones used in the food industry, the last generation’s technology and promoting an unhealthy lifestyle (smoking, eating fast food). What follows is an excerpt from an interview with a Turkish oncologist for a Romanian newspaper—an interview that was replicated on six other websites:

Fertilizers, growth hormones which increase productivity, but their ingestion has harmful consequences on genes, producing dangerous mutations with oncological potential. The pollution, including here that associated with the latest technology (smartphones, tablets) can increase the frequency of childhood cancers (leukaemia, brain tumours, some types of sarcoma)

[77].

The following excerpt is from an article in which the general suggestion is that the number of childhood cancers increased in Constanța because of the negative urban characteristics. The opposition with a rural uncontaminated realm latently articulates this narrative.

According to the doctors, poor nutrition, pollution and the high level of radiation are the main factors that contribute to the onset of the disease in the case of children. At Constanța, most of the patients are from the urban area

[86].

In fact, beneath this orchestration of guilt and responsibility in Romanian discourses lies more general anxieties produced and fed by a global, postmodern sensitivity. A very brief analysis of recent Romanian pop-culture articles dealing with different diseases reveals that the causes or triggers of childhood cancer are considered to be the same as those of cancer in general, of cardiovascular diseases and of infertility. However, what distinguishes the Romanian discourses on childhood cancer is the perception of childhood cancer as a self-inflicted wound, something that society does to itself, to its offshoots, to its most promising part—the children—who, as said in many articles, have no fault for what it is happening to them. This characteristic has to do not only with the over-visibility and social dimension of childhood cancer, but also with the idealisation of children in Western societies [3,34,59] and especially with the reverberation of this idealisation into Romanian social imagery, which is still highly indebted to a religious, traditional view of the child as a divine gift and as a path of redemption (especially for mothers) [88]. It should be noted, however, that an extensive study found that if people with cancer are socially stigmatised, children with cancer are, on the contrary, idealised in English-language magazines in North America [89]. The primary reason for this idealisation seems not to be related to a Romanian specific but, rather, to the general contemporary tendency to see children in a positive light [3,34,47,48,59].

By comparison, representations from other countries insist more on genetic mechanisms than on environment and lifestyle, emphasising to a greater extent than the Romanian representations the rarity of the disease: “Cancer in children is comparatively rare; when it does occur, it is more likely to have been triggered by something in the child’s genetic makeup than by anything to do with lifestyle or the environment” [83]. Moreover, in the UK articles that we explored, when they deal with social aspects of cancer, the comparison is made not between the UK and other European countries but between Europe and Asia or Africa: “the research suggests that the situation depends on the location (…) The researchers say around 92% of all new childhood cancer cases occur in low- and middle-income countries, with a particularly high rate in the countries of western Africa” [82]. This suggests a different approach to the problem: the social dimension of cancer is assumed not in terms of blaming Western society, nor in terms of overpraising it for its medical achievements, but as a more detached way of seeing the contextual variability and the powers of the social element. 

### 3.2. ‘All to Blame’: Making Chidlhood Cancer a National Illness

However, blaming society is not the only salient tendency that structures the tone and topics of the Romanian discourses dealing with childhood cancer. The tendency to transform childhood cancer into a sort of national disease might prevail in terms of frequency, especially since the project of the Give Life Association began to benefit from media interest and exposure. Moreover, a series of national medical and political problems are brought into attention, often by using the same emotional voice as is used for the emphasis on the social dimension but directing it towards a more virulent and caustic register, many times discussing well-acknowledged issues in Romanian contemporary popular culture [90] that have been partially documented by academic studies [91]. These problems are presented either as triggers or as other sorts of explanation for how childhood cancer is lived, understood and managed by Romanian society. As a matter of fact, the medical issues can also be viewed as a specific subset of the political ones—the very first manifestation and the most visible point of a political system perceived as viscerally corrupt and immoral. 

#### 3.2.1. The Blame on the Healthcare System

The Romanian healthcare system, underfinanced by all Romanian political regimes [90,92], is blamed for the fact that childhood cancer has a more negative prognosis than in other countries. Over the past year, news platforms such as HotNews, offered a considerable space to discuss in a very detailed and informed manner the problems of the medical system that affect the conditions in which young patients are diagnosed and treated [93,94]. Moreover, the website of the Give Life Association is replete with articles and televised debates that deal extensively with this topic, although from the perspective of the founding members of the Association—the two women who initiated the project of building a paediatric oncological hospital and more in the scope of promoting the project and its fundraising activities. Many of the discussed medical problems relate to the poor quality of life of the suffering children and of their families, determined by the improper conditions in Romanian hospitals and other medical establishments. Another portion are concerned with more specific aspects revolving around medical diagnostics and treatments that, to a large extent, fail to be successful because of what is believed to be a perverted system where doctors are sometimes indifferent or too overwhelmed to act professionally and ethically and where resources (drugs, access to medical instruments or procedures) are lacking or misallocated due to the selfish interests of those with (political) power.

In the discourses of the founders of the Association and also in the news and articles that present their vision devotedly, there is a focus on the hospital as the basic medical unit where life (and children especially, as the most condensed form of life—as a promise of a future life), cancer and death are all sheltered in different proportions. Ideally, hospitals should be those places where life triumphs over death by untying the unjust connections between children and cancer. The founders of the Association aim for this kind of hospital and stress that it is not be possible without an adequate infrastructure that serves both the quality of life of patients and their families and the medical act as such. Furthermore, there should be also a sufficient number of physicians, carriers of a new mentality, who will not deprive patients or their caregivers of agency. A new mythology of the hospital emerges—the good, desired, powerful hospital that the NGO wants to construct will be in itself a form of battle against a healthcare system that offers only hospitals where health is only a theoretical, neglected right and for which children and caregivers have to fight: “In our country the children diagnosed with cancer fight not only against the disease but also against the conditions of the healthcare system. They stay locked in for months in a hospital saloon which becomes for them their second home and they wait for their turn to the toilette and take a shower” [95].

In his recent anthropological study of a Romanian oncology hospital, Vintilă Mihăilescu [91] brings to the forefront the particularities of the Romanian healthcare system, identifying the dialectic tension between misery and miracle. Misery refers to poor conditions and problematic access to treatments and diagnostics—and indeed, the Romanian healthcare system is the weakest in Europe [96]—signalling that a miracle needs to happen in order to stop the misery from proceeding to its last consequence (which is death), disobeying order and defying predictability. It is not that miracles should not exist, but as Mihăilescu observes, they should not be essential in negotiating between illness and health, between cancer and death: “Health should not be related to, it is not allowed to depend on miracle (…) or this is what happens the most in Romania: for too many times, our health, as much as it is, as it is, seems to be about miracle” [91] (p. 19). Rather than encouraging a medical system where miracles play an important yet confusing role, the Romanian anthropologist seems to denounce both what creates the need for a miracle and how the miracle is employed to make sense of illness in a medical context. 

Similar to this idea of a miracle, but also having some distinct traits that deserve to be examined, is the concept of ‘magic’ that infuses a great part of the discourse surrounding childhood cancer. It is a keyword in the fundraising campaign of several projects of the MagiCamp Association, which defines itself as aiming to offer children with cancer the possibility of spending a joyful time in well-organised camps (“to have fun, under specialised medical supervision, is our main goal” [97]). MagiCamp also develops other related projects: MagicHome (houses near oncology hospitals, accommodating parents of hospitalised children) and MagicBox (boxes with staple foods, clothing, footwear, personal hygiene, etc. for children undergoing cancer treatment and in vulnerable economic situations). “Together we make magic happen” is one of the mottos of the MagiCamp Association. This togetherness is directed against a healthcare system seen as instrumentalised by corrupt politicians for their benefits. Magic combines three layers. First, the layer of irrationality: as rationality has failed to manifest care for others, there is a revival of the sentiment in these recent Romanian representations of childhood cancer, without any appeal to a sacred transcendence. Second, the layer of fictionality: an attempt to resort to the power of story—of a fable—as a means to recreate reality. Third, the layer of the unnatural: where death, as a natural phenomenon, is weakened. In this sense, those who make magic happen—people who donate or who volunteer for these projects—not only fight against the system, but also, most importantly, give back a childhood to the children with cancer (a frequent expression on MagiCamp websites). They disconnect, even if only temporarily, cancer from dying and children from cancer. Magic is used to restore what can be called the ‘endangered’ transcendence of the child, attacked by illness and by the grim prospect of death. Because the death of the child is culturally assigned, as a responsibility, to society, it is the society that must restore, through magic, the promise that the future can still be bright.

In comparison with media articles, especially when documenting their entire activity, the way in which MagiCamp, the Give Life Association and YuppiCamp approach childhood cancer appears to be far more complex, with a keen eye on the manifold social aspects of being an oncological patient and also a child. They address the relation between cancer and childhood, emphasising how cancer and hospitalisation steal the joy of living, limiting opportunities to spend time with friends and to explore the world, as well as the negative impact that childhood cancer has on families and caregivers. They focus extensively, however, on the side of life, while neglecting mortality as an ontological dimension (where grief is already imprinted as a response to the potential death of the Other) that intensifies under the presence of a possibly terminal illness. Let us not forget that, somehow paradoxically, it is this very incurability—not naturally but due to poor social management—that is emphasised by the Romanian discourses on childhood cancer. The positive outlook on childhood cancer and the pragmatic orientation of the actions initiated by these NGOs are, by no means, aspects that should be ignored or deemed superficial. For example, studies have revealed that indeed, recreational or therapeutic camps for children with cancer do have a positive impact on elements such as mood, friendship and quality of life [98]. Many camps for children with cancer in other countries include psychological counselling and programs for learning coping skills and accepting death as a possibility [99]—an indication that considering death could be a reason for their success.

If we agree with the idea that the hospital is a reflection of society [91]—a quintessence of society’s mechanisms of distributing roles and shaping power relations—then we will see that the project of the Romanian NGO is an attempt to change Romanian society. The same can be said about the démarches made by organisations such as MagiCamp and YuppiCamp to install magic in the middle of an ugly world where children die and the state and its healthcare system seem to be careless. On a symbolic level, it is an attempt to get rid of death and tame childhood cancer. This is a taming produced by incorporating childhood cancer into the strategies for making it more tolerable for children and society and by concealing it within the sense of empowerment that one could gain from it.

#### 3.2.2. The Blame on the Political System

The problematic healthcare system is only a reflection of a state in which particularities are engendered by a corrupt political system unwilling to and incapable of designing good social and medical policies and whose actions have contributed to a chronic state of poverty for an important segment of the population, with poverty being linked to cancer by medical studies [63,100] and public opinion as well [101]. There are many illustrations of the negative outcomes of such a disinterest in the Romanian representations of childhood cancer from recent years: from the scarcity of hospitals and poor or limited access to cancer care (chemotherapy, radiotherapy and other procedures) to terrible conditions in hospitals, both for patients and for doctors [74,88,93,94]. If the blame on the political system for the way in which childhood cancer is managed in Romania is often encountered in news and articles presenting information about childhood cancer, the discourses produced either by the Give Life Association or those revolving around its activity and mission are structured, in terms of lexical metaphors and semantic content, by the narrative of the fight against the State:

In the context of a Romanian state that in the latest 50 years did not build anything in the domain of pediatric oncology, The Give Life Association has taken on the mission to erect the first hospital of Oncology and Radiotherapy for children diagnosed with cancer in Romania. It is for the first time in Romania when an NGO builds a hospital from scratch, exclusively from donations and sponsorship

[102].

Instead of taking care of its people, the state does much worse: it condemns its children. As the children are a state’s future, symbolically, this is equivalent to a self-condemnation—to a ‘national’ self-inflicted wound. Consequently, a new rhetoric emerges—that of fighting back against the state, which means remediating, as much as possible, the ‘national’ harm: “When the state condemns sick children, it is our duty to save them” [103]. In this dissociation between a state, fallen prey to corruption, and the citizens who still believe in their power to change the world, especially when children—social entities highly infused with positive meanings—are at stake, lies the hope that death can be cured, but also the suggestion that children’s death is a matter of adults engaged in measuring their powers and confronting one another, which somehow thus denies the death of a child. 

Another reason to observe and to analyse this rhetoric is its divergence from the general narratives widespread in the Romanian media and public opinion when illness and death are on the spot. Consider, for example, a very significant event from 2015—the deadly fire at the Colectiv nightclub that killed 64 people and for which the government decreed three days of national mourning [104]—and a very recent event, the abduction and murder of a 16-year-old girl in 2019 [105]. In both cases, local authorities and institutions—such as the police (criticised, for example, for taking more than 19 h to locate the young girl)—the Orthodox Church, the Ministry of Health, etc.) were identified as moral culprits and blamed for not doing their jobs and not respecting deontological protocols. By extension, the entire state was held responsible for promoting corrupt people both in elevated and normal positions in all its institutions. In the case of the Colectiv fire, the then prime minister resigned due to the many street protests all over the country. Although the people and media railed against the state and identified corruption as a national disease, many voices argued for the acceptance of a collective guilt and a public penitence. ‘We are all to blame’ became a sort of mantra for both events [106,107]. We could not find similar declarations and stances in childhood cancer articles, which is strange, considering that in many social circumstances where children appear as vulnerable and that have a potential to be interpreted in a political register (poverty in schools, child murders, etc.) this collective guilt emerges. An explanation could be that cancer is still perceived as a negative force with which no one wants to be associated—something, perhaps, on an imaginary level, even contagious. Cancer may be an element that, despite being over-visible in terms of its social dimension, is still feared as a whim of biology, of nature or as the face of death. 

Returning to the images of the fight, the result of a war-like situation in which the state fights its citizens and the citizens fight the state, is that death is eliminated, reduced to an object of dispute. For a better understanding of this part, we can also use the theoretical framework proposed by Adela Toplean, who explored extensively the relationships between death and the state, observing that a salient tendency of contemporary social Romanian attitudes and discourses is to associate death and corruption to the point of the two becoming inseparable: “death in Romania is inseparable from corruption” [108,109]. The consequences are that, from a general perspective, “nobody, in our country, dies from death, but from corruption” [110] and from an individual point of view, “nobody, in these situations, does not feel they *owe* a death (because nothing in the ways in which the world is socially, spiritually, existentially and culturally established does justify such a duty)” [109]. Moreover, not only our social and personal connections with death are obliterated, but there is also “a symbolical depreciation, increasingly marked, of death itself” [110]. The ‘institutional algorithms of un-blaming’, says Toplean [110], might be a too convenient strategy for the individual not to feel responsible for their own ontological dimension of death and the death of the Other, as an interpersonal and social event. Insisting on the ‘systemic’ dimension of death, Toplean believes, precludes real changes in the system.

In a similar way, a too intense focus on the national, ‘systemic’ aspects of childhood cancer, be they medical or strictly political, could lead to neglecting both cancer as a complex illness that needs to be addressed from an interdisciplinary and multidisciplinary perspective and to encouraging our inner impulse to deny death [55,56], not just in relation to cancer, but also in relation to life as such. Furthermore, undervaluing the symbolical side of death and making dying a matter of political power obscure the fact that children can and do die and the psychological challenges of their families and caregivers.

## 4. Conclusions

When an illness with such an agitated cultural history as cancer brings together death and children, complications arise, because, despite many signs of progress in this direction, cancer is still perceived as a terminal illness, while children, highly valued in contemporary society, function both as an immanent transcendence (for their parents and families) and as a social transcendence (for postmodern societies that cultivate on a large scale a form of symbolic immortality through children. [18,22,24]). To briefly summarise what we discussed in this paper, we will begin by saying that the representations that we investigated in Romanian media are ambivalent.

Even if imperfect in the ways in which they utilise arguments and despite their tendency towards emotional exploitation, these representations indicate—through this excess of negative images of childhood cancer, of redundancy and of simplification in blaming society and the political system—an effort to change the system and to produce a change in a country where the reality is that children do die of cancer more than in other Western countries [111] because of failures to identify treatable cases of cancer. Moreover, these representations familiarise the audience with and raise awareness of some very important social and medical aspects of the everyday reality of children suffering from cancer.

With such an approach, death as a possible result of cancer detaches completely from a normal death, no matter how painful or unnecessary—a death that should be always considered as an unfortunate possibility in any disease and for any age category. Cancer, here, does not refer to the actual disease, to a cancer as such (in which case eliminating death would be wonderful and a victory) but to its socio-symbolical layers—to cancer as a product of the contemporary social and cultural imagination. In addition, neglecting the notable social progress in managing childhood cancer [40] or disregarding the fact that it is not among the main causes of child mortality [112,113] may lead to creating a deformed image of social reality, enforcing an already imbalanced social relation between children as assets and other representatives roles, especially seniors as burdens and to losing any historical perspective on the world in which we live and our capacity to understand its social dynamics. We are witnessing a case in which the social imagery, where children and childhood are idealised and detached from their mortal vein, impacts the social relevance of a topic more than the social reality as such. As Dixon-Woods et al. observed: “One of the most striking features of childhood cancer has been the dramatic improvement in prognosis for children diagnosed with cancer. In 2001 the death rate for childhood cancer in the UK was 300 children per year. Only 30 years previously, in 1969, there were 850 deaths” [40]. At least in Romania, diseases other than childhood cancer, in terms of social relevance, seem to be more urgent. For example, although data about cancer in Romania are almost completely lacking [79], it is known that this country has the highest rates of incidence and mortality in Europe for cervical cancer, a very preventable type of cancer. 

When cancer is seen through political lenses, there is a risk of ignoring the inherent tragedy that resides in any unpreventable illness, narrowing the ontological, existential dimension of death to a strictly political one, which would bring the denial of death [55,56] if not to the forefront, at least behind the scene. For both social and political over-interpretations of childhood cancer, the risk seems to be the same as that denounced decades before by Susan Sontag [65]—that of wrapping in cultural narratives (to the point of suffocating) a disease that otherwise is part of the accidental and biological functioning of life. Moreover, if it is not questionable that there is much need for magic in the lives of children with cancer and their families, encouraging a dichotomy between magic and misery, with a reframing of magic in a paradigm of a profane, man-made transcendence, may have some benefits (connected to fundraising and enhancing a proactive attitude), but it could hinder the acceptance of the mortal nature of human beings and the limited power one has over the death of the other. 

When this magic insists on the ‘fun’ or easy side of donating (“help the children diagnosed with cancer by sending a simple SMS”) and of being involved in childhood cancer projects, it may too greatly tame the ability to care for others, leading to the corrosion of ethics and the rise of what Gilles Lipovetsky [114] calls the ‘painless ethics’ of postmodern times. It might be true that the Romanian State did little or nothing for paediatric oncology, but the same applies to other healthcare branches. Thus, creating a public enemy position for the insufficiencies that characterise the Romanian healthcare system and identifying the enemy as the state or the medical system might not be too accurate or helpful in strict relation to childhood cancer. Although making cancer a national illness has the advantage of highlighting the numerous problems in the Romanian healthcare system, including inefficient medical policies and the negative impact of corruption on how childhood cancer is socially managed may lead to overlooking the global context of the disease, minimalizing the overall progress and the achievements of modern medicine in diagnosing and treating childhood cancer. The risk in the proliferation of these kind of representations of childhood cancer is to offer sketched and ideologized images of death, which limit the understanding of human mortality as an impending reality and as a personal and interpersonal dimension, stimulating the production of stereotypes (e.g., children should not die; a child’s death is the worse death; the state is evil; the medical system is to blame; etc.) and encouraging reactive thinking to the detriment of a more subtle manner of coping and negotiating meanings in relation to one of the things we most dread, death and what we cherish and praise, children.
